# Trauma patients have reduced *ex vivo* flow-dependent platelet hemostatic capacity in a microfluidic model of vessel injury

**DOI:** 10.1371/journal.pone.0304231

**Published:** 2024-07-10

**Authors:** Kimberly A. Thomas, Rassam M. G. Rassam, Ronit Kar, Devin M. Dishong, Katelin C. Rahn, Ricardo Fonseca, Melissa Canas, Jose Aldana, Hussain Afzal, Kelly Bochicchio, Matthew D. Neal, Grant V. Bochicchio, Philip C. Spinella, Susan M. Shea

**Affiliations:** 1 Vitalant Research Institute, Denver, Colorado, United States of America; 2 Trauma and Transfusion Medicine Research Center (TTMRC), Department of Surgery, University of Pittsburgh, Pittsburgh, Pennsylvania, United States of America; 3 Department of Surgery, Washington University in St. Louis, St. Louis, Missouri, United States of America; 4 Department of Critical Care Medicine, University of Pittsburgh, Pittsburgh, Pennsylvania, United States of America; 5 Department of Bioengineering, University of Pittsburgh, Pittsburgh, Pennsylvania, United States of America; Chung-Ang University College of Engineering, REPUBLIC OF KOREA

## Abstract

Trauma is the leading cause of death in individuals up to 45 years of age. Alterations in platelet function are a critical component of trauma-induced coagulopathy (TIC), yet these changes and the potential resulting dysfunction is incompletely understood. The lack of clinical assays available to explore platelet function in this patient population has hindered detailed understanding of the role of platelets in TIC. The objective of this study was to assess trauma patient *ex vivo* flow-dependent platelet hemostatic capacity in a microfluidic model. We hypothesized that trauma patients would have flow-regime dependent alterations in platelet function. Blood was collected from trauma patients with level I activations (N = 34) within 60 min of hospital arrival, as well as healthy volunteer controls (N = 10). Samples were perfused through a microfluidic model of injury at venous and arterial shear rates, and a subset of experiments were performed after incubation with fluorescent anti-CD41 to quantify platelets. Complete blood counts were performed as well as plasma-based assays to quantify coagulation times, fibrinogen, and von Willebrand factor (VWF). Exploratory correlation analyses were employed to identify relationships with microfluidic hemostatic parameters. Trauma patients had increased microfluidic bleeding times compared to healthy controls. While trauma patient samples were able to deposit a substantial amount of clot in the model injury site, the platelet contribution to microfluidic hemostasis was attenuated. Trauma patients had largely normal hematology and plasma-based coagulation times, yet had elevated D-Dimer and VWF. Venous microfluidic bleeding time negatively correlated with VWF, D-Dimer, and mean platelet volume (MPV), while arterial microfluidic bleeding time positively correlated with oxygenation. Arterial clot growth rate negatively correlated with red cell count, and positively with mean corpuscular volume (MCV). We observed changes in clot composition in trauma patient samples reflected by significantly diminished platelet contribution, which resulted in reduced hemostatic function in a microfluidic model of vessel injury. We observed a reduction in platelet clot contribution under both venous and arterial flow *ex vivo* in trauma patient samples. While our population was heterogenous and had relatively mild injury severity, microfluidic hemostatic parameters correlated with different patient-specific data depending on the flow setting, indicating potentially differential mechanistic pathways contributing to platelet hemostatic capacity in the context of TIC. These data were generated with the goal of identifying key features of platelet dysfunction in bleeding trauma patients under conditions of flow and to determine if these features correlate with clinically available metrics, thus providing preliminary surrogate markers of physiological platelet dysfunction to be further studied across larger cohorts. Future studies will continue to explore those relationships and further define mechanisms of TIC and their relationship with patient outcomes.

## Introduction

In the United States (US), trauma is the leading cause of death in individuals 44 years of age or younger [1]. Furthermore, approximately 30,000 deaths due to hemorrhage per year in the US are preventable after injury, and occur due to delayed or potentially inadequate care [2]. A quarter of trauma patients present clinically with trauma-induced coagulopathy (TIC) [1, 3], which increases the risk of mortality by 400% [4]. TIC is a complex phenomenon induced by a variety of contributing factors, including tissue injury, shock, endothelial dysfunction, immune dysregulation, consumption of coagulation factors, iatrogenic hemodilution, and platelet dysfunction [5].

Platelets participate in hemostasis and thrombosis in a complex, multi-faceted, and flow-dependent manner. Platelets trigger rapid function responses that control not only hemostasis, but inflammatory and immune responses as well, serving as key participants in immunothrombosis [6], and are integral for maintenance of vascular integrity [7]. Importantly, they must also be able to perform these actions in all flow settings of the vasculature, including at high, and even pathologically high, flow regimes, where forces and shear stresses work to counteract adhesion. Platelets must therefore be able to adhere, activate, and interact with their environment extremely rapidly, which they do so through various receptors (reviewed in [8]), to include but not limited to GPVI [9], GPIIb-IIIa (CD41/61), GPIbα/β-IX-V (CD42B,C,A,D respectively) [10], P-selectin (CD62P), and platelet protease-activated receptors (PARs, e.g. PAR4) [11]. Activated platelets mediate the assembly of procoagulant complexes on their anionic phospholipid-rich surface, which generate the necessary thrombin burst to propagate coagulation via fibrin generation [11]. GPIIb-IIIa also contributes to platelet:platelet interactions leading to clot stabilization and retraction [12]. There are therefore many potential mechanisms by which platelet dysfunction during TIC may be occurring, and furthermore there is variability in the trauma patient population with respect to biology, genetics, and specifics of injury that will compound underlying dysregulation.

As the underlying mechanisms regulating alterations in platelet function during TIC remain poorly defined, it is difficult to both evaluate tests to measure this dysfunction as well as establish which therapeutic interventions would be most beneficial. Further complicating understanding this pathophysiology is the heterogeneity of the trauma patient population. Studies of platelets from trauma patients show widespread incidence of impaired function, yet the clinical implications are unclear [13–15]. While studies to date have clearly demonstrated measurable *in vitro* and *ex vivo* platelet dysfunction in the vast majority of trauma patients, not all trauma patients with platelet dysfunction present with concomitant clinical bleeding, making it difficult to interpret assay outputs [13]. In order to understand how platelet dysfunction contributes to clinically-relevant hemostatic dysfunction, there is a need for appropriate biofidelic assays linked to patient-specific data. The limited availability of these tools for measuring platelet function has impeded detailed study of this pathology.

In the setting of local vascular injury, shear rates are estimated to be as high as 45,000 s^-1^, akin to pathological thrombosis levels [16]. In the setting of such high flow regimes and high shear stress, specific mechanisms must be engaged to ensure capture of platelets on extravascular surfaces exposed by injury to contribute to stopping flow. In contrast, the diffusion-dominated protein bursts that drive the protease cascade of classical coagulation require very low shear stress (e.g. near stagnant, <100 s^-1^) before proceeding. Vascular maintenance in low shear regimes must also be reestablished in the peri- and post-injury phases. The paradigm of trauma coagulopathy and the respective flow settings therefore demand multiple aspects of platelet function. A holistic approach (i.e., assessing platelet function and hemostasis in both low and high shear regimes) is thus essential to obtain a complete understanding of trauma pathophysiology, and these functional capacities must be studied within the vast mélange of trauma patient subsets and endotypes (for example, recently identified inflammatory endotypes [17]).

The objectives of this study were therefore (i) to assess the hemostatic functional capacity of samples obtained from trauma patients upon hospital arrival in a microfluidic chamber simulating vascular injury at both venous and arterial shear rates, and (ii) evaluate which clinical metrics correlate with reduced hemostatic capacity under flow conditions. We hypothesized that trauma patients would have differential flow-dependent alterations in platelet function at venous and arterial shear rates.

## Materials and methods

### Blood sample collection

Samples were collected in sodium citrate vacutainers from adult trauma patients (N = 34) with Level I trauma activations within 60 min of arrival to the Emergency Department (Washington University in St. Louis Institutional Review Board (IRB) protocol #202010174, written consent obtained, recruitment dates 10 November 2020–13 Aug 2021). Level I activation requirements are institution-specific and are listed in S1 Table. While our analysis does not control for resuscitation that may have taken place prior to collection, no prehospital transfusion of blood products occurred as it was not available during the sample collection period. Patient data was collected from medical charts including demographics, mechanism of injury, injury severity, hemodynamic parameters, blood products transfused, transfusion reactions, surgeries or other interventions performed, medications administered, and mortality. Samples were also collected from healthy volunteers (N = 10) as controls (University of Pittsburgh IRB protocol #21100141, written consent obtained, recruitment dates 18 February 2022–31 April 2022). For all samples, complete blood counts were collected using an Advia 2120i (Siemens, Munich, Germany) prior to use in microfluidic assessment and further processing to generate platelet-poor plasma (PPP). Demographic information was not collected from healthy adult volunteers. This population was recruited from a generally young healthy population at the University of Pittsburg.

### Microfluidic assay

**Fig 1:**
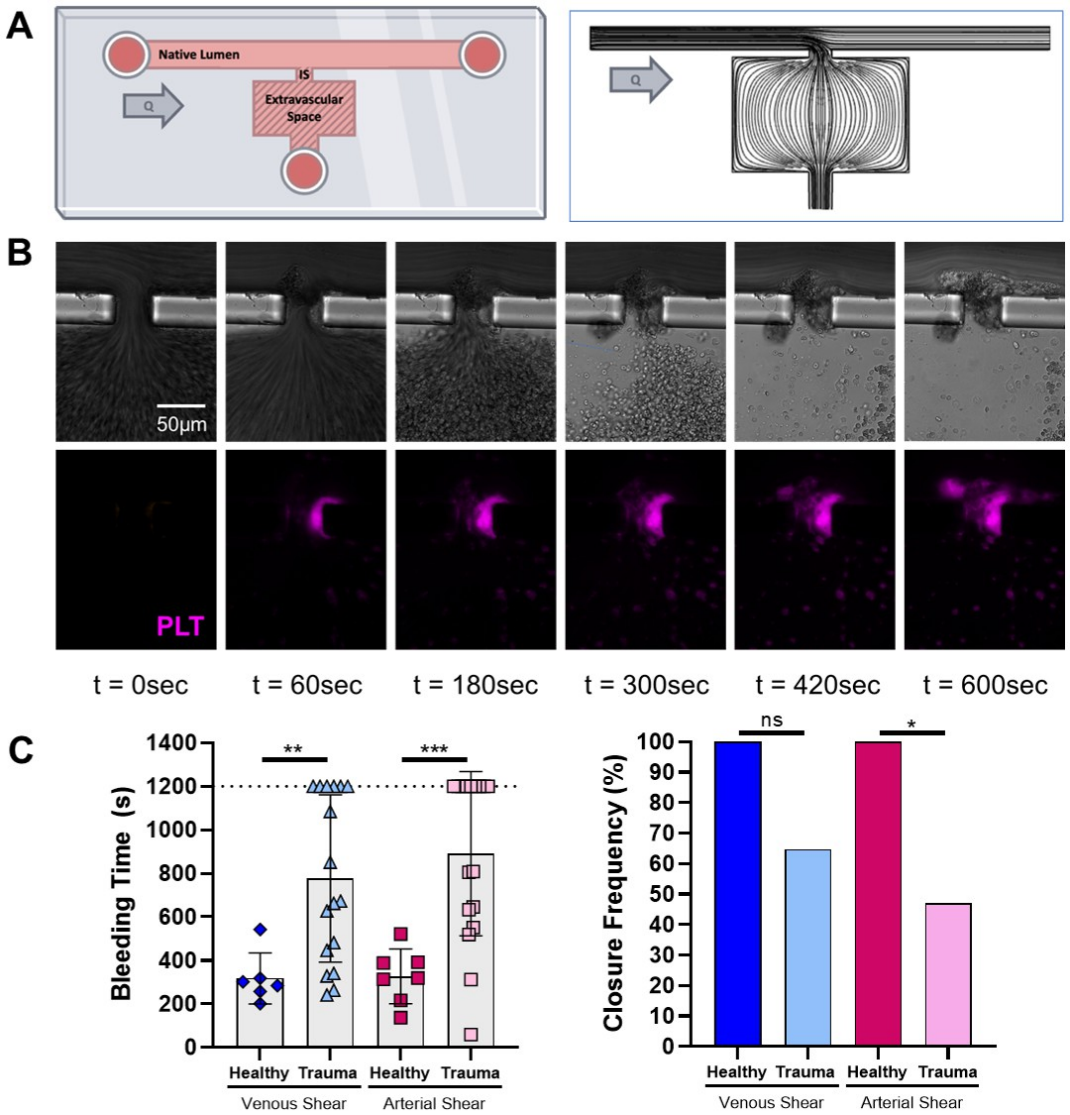
Trauma patients had significantly increased bleeding times in a microfluidic model of vessel injury. **A** The microfluidic model comprises a native lumen, injury site, and extravascular space. Streamlines from simulated flow are shown. Healthy controls form a clot at the injury site resulting in microfluidic hemostasis and generation of a bleeding time (BT). **B** Representative control microfluidic hemostasis timelapse. Transmitted light (top) and platelet (anti-CD41) fluorescence (bottom, pink). **C** Left, samples from trauma patients had longer BT compared to healthy control samples at both venous and arterial shear rates when perfused through the microfluidic devices. Right, while 100% of the healthy control samples clotted, trauma patient samples had reduced closure frequency compared to healthy controls. Bleeding time are shown as individual values with bars representing mean and error bars representing standard deviation. Closure frequency is shown as absolute value. ns: not significant, * p<0.05, ** p<0.01, *** p<0.001.

The microfluidic chamber simulates vascular injury (Fig 1A). The chamber (50 μm height throughout) comprises a native lumen (150 μm width), an injury site (50 μm width), and an extravascular space (>5x injury site width; Fig 1A). Flow through the chamber was modeled using COMSOL (version 5.5, COMSOL, Inc., Burlington, MA, USA), assuming whole blood as a Newtonian fluid (3.5 cP) and flow presumed as laminar, incompressible, steady, continuous, and isothermal (convergence criterion: residual values<1e^-6^). The chamber was modeled with no-slip wall conditions with a fully developed flow inlet, and a zero-pressure outlet, reflecting the experimental setup. Resulting streamlines are shown in the right of Fig 1A, and simulations determined inlet flow rates that resulted in the target perfusion initial wall shear rates. Molds were made using photolithography in the Washington University in St. Louis Institute of Materials Science and Engineering (IMSE) clean room. Polydimethylsiloxane was cured over the mold for 3 hr at 70°C to create the negative shape of the chamber and plasma-bonded via a benchtop plasma cleaner (Harrick Plasma Inc, Ithaca, NY, USA) to a glass slide, and chambers were then rinsed with ethanol and dried at 70°C overnight. The extravascular space and injury site were coated with collagen (ChronoLog, Havertown, PA, USA) and tissue factor (Dade Innoven Reagent, Siemens, Munich, Germany) via overnight incubation with a 7:2:1 sodium chloride:collagen:tissue factor solution. Prior to experimental use, channels were then rinsed with phosphate-buffered saline (PBS) and the native lumen incubated with 5% bovine serum albumin (BSA) in PBS for 30 min, followed by another PBS rinse. Samples were perfused via syringe pump through the bleeding chamber at native lumen wall shear rates of either 150 s^-1^ or 3500 s^-1^. Calcium chloride (100 mM; Millipore-Sigma, St. Louis, MO, USA) was perfused immediately upstream of the testbed at 10% of the sample perfusion flow rate to achieve a target final concentration of 10–15 mM for sample recalcification. Samples were perfused until the chamber achieved occlusion of the injury site and flow out of the native lumen was stopped for at least 3 min. The time from initial perfusion to injury site occlusion was defined as the bleeding time (BT; s). If no seal was achieved, the assay was stopped at 1200 s (20 min). The microfluidic assay was performed on samples from 20/34 trauma patients.

For a subset of microfluidic experiments (N = 6 trauma patients, N = 4 healthy controls), samples were incubated with fluorescent anti-CD41 (#NB100-2614, used at 1:600; Novus Biologicals, Centennial, CO, USA) and Human TruStain FCX (1:200, to block non-specific FcyR:immunoglobulin interactions; #422302, BioLegend, San Diego, CA, USA) for 30 min prior to perfusion.

### Image acquisition and analysis

Images of the injury site in microfluidic assays were acquired using an AxioObserver 7 inverted microscope, Axiocam 506 camera, and Zen software version 3.6 (Zeiss, Munich, Germany). For quantification of mean fluorescence intensity (MFI), raw images were exported and intensity quantified via summation of image matrices in Python (version 3.9, Python Software Foundation, Wilmington, DE, USA). For quantification of surface area, brightfield images were exported and clot pixels manually segmented in Photoshop (version 23.2). Representative kinetic images in healthy controls are shown in Fig 1B. Pixels were converted to surface area using the conversion factor recorded by the microscope software. Lag time and growth rate were extracted using MATLAB (version R2021a; Mathworks, Natick, MA, USA), by first applying a LOESS function to smooth noise and then identifying points of inflection. Slopes between points of inflection were calculated using a central difference (three point running average) and classified as lag if the absolute value of the slope was >1.5 μm^2^/s, which was established using a training set of 5 randomly selected experiments.

### Plasma-Based assays

Blood samples were centrifuged at 1600 *g* for 10 min followed by 2500 *g* for 10 min to generate PPP, which was then immediately aliquoted and frozen at -80°C. At the time of assay, PPP was thawed at 37°C for 5 min and assayed for prothrombin time (PT), partial thromboplastin time (PTT), fibrinogen (FGN), VWF, and D-Dimer using a Stago Compact Max coagulation analyzer (Diagnostica Stago, Parsipanny, NJ, USA) per manufacturer’s instructions.

### Statistical approach

Clinical and epidemiological data were reported as N (%) or median (interquartile range (IQR)). Other data were visualized and analyzed using GraphPad Prism (version 9.5.1; GraphPad Software, San Diego, CA USA), and are shown as individual points with lines at mean and error bars showing standard deviation (SD), unless otherwise indicated. Unpaired t-tests were used to compare groups (α = 0.05). Data from trauma patients, to include clinical and hemostatic function data, were subjected to a correlation analysis using Pearson’s R to identify relationships for future hypothesis generation.

## Results

Patient demographic and injury data is summarized in Table 1. Patients were majority male (76%) and had mostly blunt injury (65%). The median injury severity score (ISS) was 10. Only one patient did not survive to discharge.

**Table 1:** Trauma patient characteristics.

***Patient Descriptors***	*n (%) or median (IQR)*
Sex	
Male	26/34 (76%)
Female	8/34 (24%)
Injury	
Blunt	22/34 (65%)
Penetrating	12/34 (35%)
Race	
White	12/34 (35%)
Black or African American	20/34 (59%)
Other or Unknown	2/34 (6%)
Age	36 (24–58)
BMI	25.4 (23.6–30.7)
***Injury and Critical Illness Severity***	*n (%) or median (IQR)*
Admission GCS	15 (13–15)
ISS	10 (9–24)
APACHE II	12 (9–18)
Received Any Transfusion	11/34 (32%)
Survived to Discharge	33/34 (97%)

Trauma patients were majority male, had majority blunt injury, and were majority black or African American. The cohort had largely low/moderate injury severity. BMI: body mass index, GCS: Glasgow Coma Scale; ISS: injury severity sore; APACHE II: Acute Physiology and Chronic Health Evaluation, an intensive care unit (ICU) mortality prediction score.

**Fig 2:**
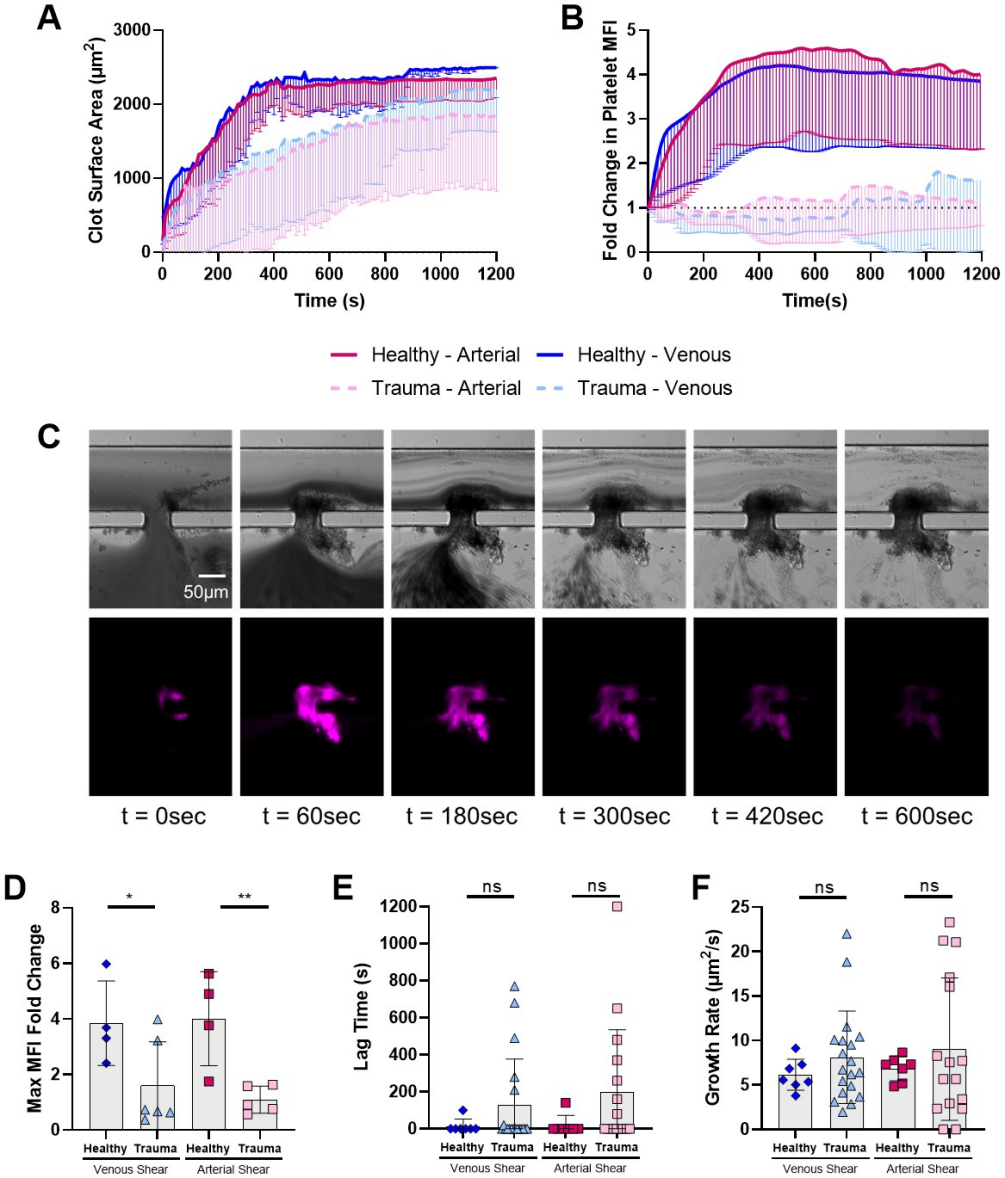
Despite similar amounts of clot deposited, trauma patient samples have massively reduced platelet contribution to microfluidic clot. **A** Trauma patient samples had slightly reduced clot surface area deposition but overall deposited substantial amount of clot at the injury site. **B** Trauma patient samples had massively reduced platelet deposition compared to healthy controls at both venous and arterial shear. **C** Representative trauma microfluidic hemostasis timelapse from a sample that achieved hemostasis. Transmitted light (top) and platelet (anti-CD41) fluorescence (bottom, pink). **D** Maximum platelet MFI fold change was significantly reduced in trauma patient samples. Traces are shown as mean lines with standard deviation indicated as error bars below. **E, F** Lag time and growth rate of total surface area deposition were not different between trauma and controls. Individual data points are shown with bars at mean and error bars indicating standard deviation. ns: not significant * p<0.05, ** p<0.01, *** p<0.001. MFI: mean fluorescence intensity.

In the microfluidic model of injury, trauma patients had longer microfluidic BTs than controls for both venous (mean±SD; 776±93 s vs 317±48 s; p = 0.010) and arterial (890±92 s vs 327±47 s; p<0.001) shear rates (Fig 1C). Healthy controls always resulted in complete hemostatic closure of the microfluidic injury site, while in trauma patient samples only 65% (venous) and 43% (arterial) resulted in hemostatic closure (Fig 1D). Significant differences were not observed in bleeding times at either shear rate when comparing polytrauma patients (ISS>16) to minor trauma patients (ISS<16) (S1 Fig). Despite end total clot deposition achieved at both venous and arterial shear rates and similar platelet counts, the platelet deposition was massively attenuated in trauma samples (Fig 2A and 2B) and the maximum MFI fold change was lower in trauma samples at both venous and arterial shear (Fig 2D). Kinetic images experiment with clot and bleeding time generated is shown in Fig 2C, demonstrating initial deposition of platelets that do not remain in the clot. Clot deposition was variable in trauma patients and a small subset patients had little-to-no deposition at all in the model. Thus, trauma patient samples also had slightly longer lag times, though this was not statistically significant, and wildly variable growth rates, yet the mean growth rate was comparable to healthy samples (Fig 2E and 2F).

**Fig 3:**
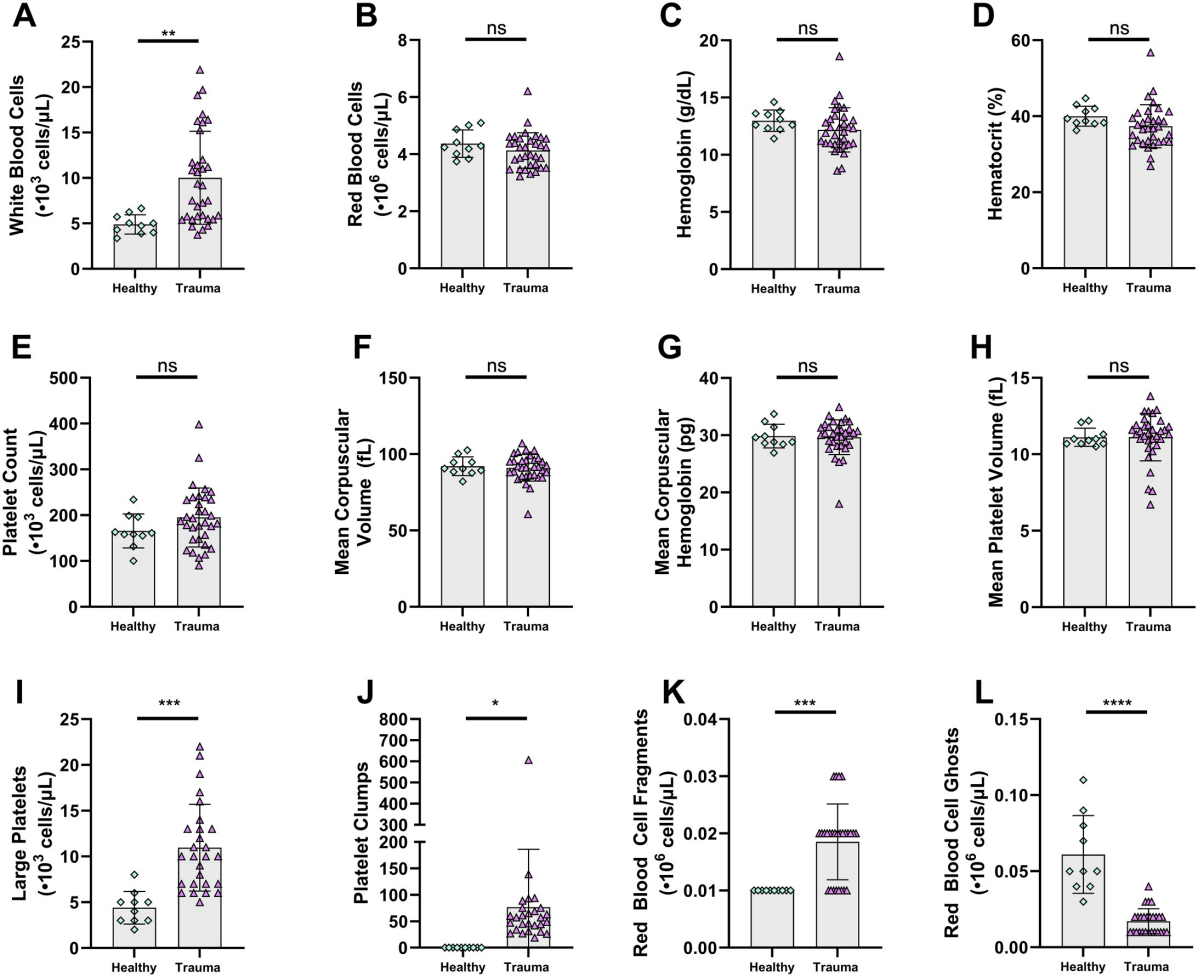
Trauma patients have largely similar hematology to healthy controls. **A-H** Complete blood count parameters indicated largely similar values in trauma patients and healthy controls, with only white blood cells elevated. **I-L** The Advia 2120i reports additional nontraditional parameters regarding platelet size and cell status. Trauma patients had elevated large platelets, platelet clumps, red blood cell fragments, and reduced red blood cell ghosts. Data are shown as individual values with bars representing mean and error bars representing standard deviation. ns: not significant, * p<0.05, ** p<0.01, *** p<0.001.

**Fig 4:**
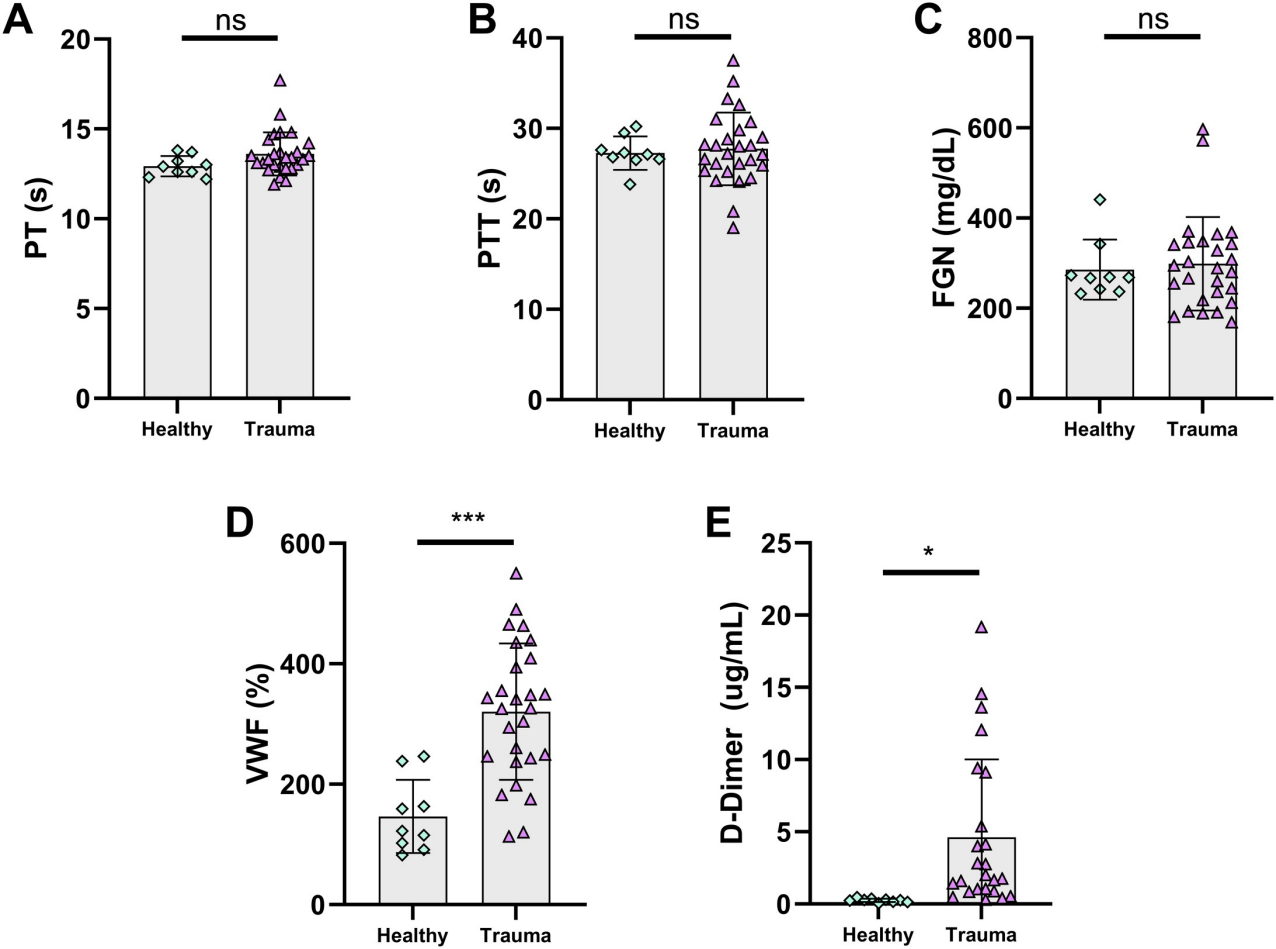
Plasma-based coagulation assays did not detect differences between trauma patient samples and healthy controls. **A-C** PT, PTT, and FGN were not different between trauma samples and healthy samples. **D, E** VWF antigen was significantly elevated in trauma patient samples, as was D-Dimer. Data are shown as individual values with bars representing mean and error bars representing standard deviation. ns: not significant, * p<0.05, ** p<0.01, *** p<0.001. PT: prothrombin time, PTT: partial thromboplastin time, FGN: fibrinogen, VWF: von Willebrand Factor.

Trauma patient samples had higher white blood cell counts (10.02±5.12·10^3^ cells/μL vs. 4.89±1.06·10^3^ cells/μL, p = 0.003), but red blood cell counts, hemoglobin, hematocrit, and platelet counts were all similar between groups (Fig 3A–3H). Trauma patient samples had significantly higher amounts of large platelets, platelet clumps, red blood cell fragments, and fewer red blood cell ghosts counts (Fig 3I–3L). Trauma patient samples had similar PT and PTT to controls (Fig 4A and 4B). FGN was also similar, though VWF was elevated in trauma samples, as was D-Dimer (Fig 4C–4E). Complete blood count parameters compared by samples that achieved hemostatic microfluidic closure vs. those that did not were similar (S2 Fig).

**Fig 5:**
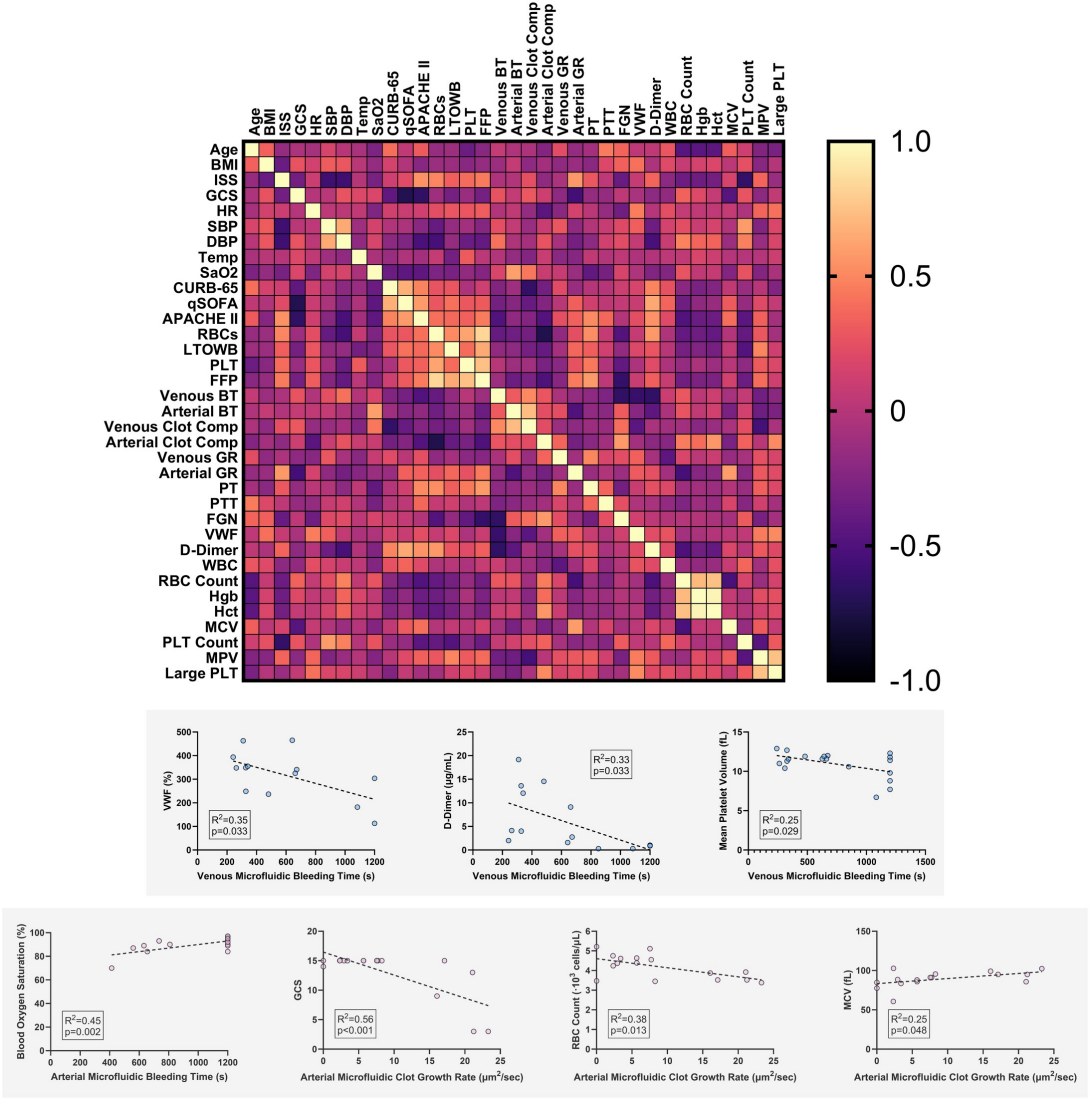
Correlation analysis revealed flow-specific signatures related to microfluidic function. **A** The correlation matrix indicates positive (yellow) and negative (dark purple) Pearson’s R values. **B** Correlations that were significantly related to microfluidic parameters. BMI: body mass index, ISS: injury severity score, GCS: Glasgow Coma Scale, HR: arrival heart rate, SBP: arrival systolic blood pressure, DBP: arrival diastolic blood pressure, temp: temperature, SaO2: oxygen saturation, RBCs: amount of red cell transfusion received, BT: bleeding time, GR: growth rate, PT: prothrombin time, PTT: partial thromboplastin time, FGN: fibrinogen, VWF: von Willebrand Factor, WBC: white blood cells, RBC: red blood cell, Hgb: hemoglobin, Hct: hematocrit, MCV: mean corpuscular volume, MCH: mean corpuscular hemoglobin, MCHC: mean corpuscular hemoglobin concentration, PLT: platelet.

To investigate relationships between microfluidic functional parameters and available clinically relevant metrics, a correlation analysis was performed on the patient demographic and injury information, clinical metrics collected at hospital arrival, complete blood count data, plasma-based assays, and microfluidic data, generating a correlation matrix (Fig 5A). Venous microfluidic bleeding time significantly negatively correlated with VWF (R^2^ = 0.35, p = 0.032), D-Dimer (R^2^ = 0.33, p = 0.033), and MPV (R^2^ = 0.25, p = 0.029), while arterial microfluidic bleeding time significantly correlated with blood oxygen saturation (R^2^ = 0.45, p = 0.002), and arterial microfluidic growth rate with GCS (R^2^ = 0.56, p<0.001), RBC count (R^2^ = 0.38, p = 0.013), and mean corpuscular volume (MCV; R^2^ = 0.25, p = 0.048). These correlations are shown in Fig 5B. The same parameters widely did not correlate with PT nor PTT (S3 Fig), with the only significant correlations between PT and GCS (negative correlation; R^2^ = 0.26, p = 0.007) and PT and D-Dimer (positive correlation; R^2^ = 0.20, p = 0.031), both of which were weaker than correlations with microfluidic parameters.

## Discussion

Using a microfluidic chamber mimicking vessel injury in both venous and arterial shear regimes, we observed elongated bleeding times in trauma patient samples compared to healthy controls, indicating reduced hemostatic functional capacity. Trauma patient samples deposited substantial amounts of clot at the microfluidic injury site when comparing surface area, yet these clots contained relatively reduced platelet content, indicating a critical role for platelets in establishing hemostasis beyond clot deposition. Plasma-based coagulation measures of coagulation (PT, PTT) did not indicate functional deficits. Sample platelet counts and other hematology parameters were the same between healthy controls and trauma patient samples, indicating reduced functional capacity and not reduced availability in circulation was responsible for differences in microfluidic hemostatic capacity.

Studies of platelets from trauma patients show widespread incidence of impaired function, yet the clinical implications remain unclear. Using aggregometry, Kutcher et al. found 46% of patients (N = 101, mean injury severity score (ISS) of 23.2) had sub-normal aggregation to at least one agonist at admission, and 91% of patients had a sub-normal response at some point during their intensive care unit (ICU) stay [13]. They also found associations of platelet hypofunction with as high as a 10-fold increase in early mortality. Vulliamy et al. recently reported profoundly reduced platelet adhesion to collagen under flow (1000 s^-1^) in patient samples (N = 39; median ISS 34), and reduced aggregation and activation responses to collagen, but normal responses to adenosine diphosphate (ADP) [14]. They hypothesized that surface receptor shedding is a possible mechanism of TIC-induced platelet dysfunction and found decreased platelet surface expression GPVI and increased presence of soluble GPVI. Li et al. found 70% of trauma patients (N = 15, median ISS 7.5) had defects in adhesion to collagen under low flow conditions (100 s^-1^) [15]. Interestingly, they found that 15% of patients exhibited a low shear microfluidic *hyper*-response.

There have been limited studies under flow, including the studies performed by Li et al. and Vulliamy et al. discussed above. These studies have been performed in vastly different patient cohorts, and also observed similar weak relationships with ISS and platelet function in low/mildly injured patient cohorts. Li et al. observed no correlation with ISS [15]. In the study by Vulliamy et al., patients with ISS>25 did have elevated levels of shed receptors, which was not observed for patients with ISS≤25. As our cohort was relatively mildly injured, our results are consistent with these previous findings.

With the objective of investigating relationships between microfluidic hemostatic functional parameters and patient and other assay data to generate the next set of hypotheses and identify potential surrogates of platelet dysfunction under flow, a correlation analysis was performed. Reduced D-Dimer correlated with slower venous microfluidic bleeding time, as did VWF. We also observed increased VWF in the trauma patient population relative to healthy controls. Plautz et al. have also reported an increase in VWF antigen levels and ultralong VWF in trauma patients, coupled with a reduction in ADAMTS13 level and activity, ultimately contributing to a shift to a prothrombotic state and deposition of microthrombi [18]. Consistent with this observation, we suspect a massive release of VWF due to platelet and endothelial activation in trauma patients results in a pathologically high amount of circulating ultralong VWF, resulting in increased venous hemostatic function. Notably, despite high levels of VWF and normal platelet counts, we still observe increased bleeding times at both shear rates, and attenuated platelet participation in microfluidic hemostasis. Consistent with the observations by Vulliamy et al. [14] this suggests loss of or reduced function of the GPIb-IX-V receptor complex, as well as potentially decreased fibrinogen binding and/or paracrine activation. We will further investigate these mechanisms and pair such analyses with functional data to increase our understanding of the TIC platelet phenotype in future studies.

Interestingly, high shear microfluidic bleeding times and hemostatic kinetics significantly correlated with red blood cell-related parameters in a surprising pattern. Reduced oxygenation was correlated with faster bleeding times, and reduced red cell count was associated with faster growth rates. Others have identified a conversion to a prothrombotic state due to hypoxia [19], identifying activation of the procoagulant pathway in low-oxygen states [20, 21]. Plasma-based coagulation assay clotting times widely did not correlate with the same parameters that significantly correlated with microfluidic parameters, indicating a potential unique ability of a microfluidic assay to identify novel important aspects of patient pathophysiology related to hemostatic function.

Beyond impaired receptor function, additional mechanisms of platelet dysfunction may be related to intra-platelet changes. Verni et al. found that total calcium mobilization was reduced in every trauma patient (N = 16; median ISS 24.5) at admission and up to 12 hours post-admission, and that while mobilization increased over time, only 25% of patients demonstrated a return to normal levels within 120 hours [22]. Fields et al. recently observed changes in the platelet transcriptome in trauma patient samples that segregated by injury type in a principal component analysis [23]. Marrying these approaches with functional metrics may provide further future insight.

Our study is limited by being dominated by low/moderate injury severity patients. However, platelet dysfunction has been previously identified as widespread and alterations in platelet phenotype and function specifically have been shown in relatively uninjured patients. We plan to continue this work and seek functional signatures related to patient outcomes, as the lack of a meaningful platelet function test remains a major barrier in this field.

Our study is further limited by being observational, and our correlation analysis is purely exploratory and intended to generate hypothesis for future and continued mechanistic investigation. While our preliminary analysis shows no difference in microfluidic bleeding time in polytrauma vs. minor trauma patients, our sample size prohibits in depth investigation of differences in these patient cohort, and more data from a larger patient cohort is needed to fully inform on differences in patient populations and clinical outcomes. Future comprehensive receptor phenotyping would also allow for comparison to findings from other studies and identification of additional receptors that may be involved in TIC-associated platelet dysfunction.

## Conclusion

We observed flow-dependent reductions in *ex vivo* platelet hemostatic capacity in trauma patients in a microfluidic model of injury. Despite apparent compensatory clot deposition in trauma patient samples and normal platelet counts, microfluidic clots were specifically lacking in platelet content indicating a critical role for platelets in our model of hemostatic closure. Protein content appears to be related to *ex vivo* venous shear function in our model, and red blood cell count and oxygenation status may be related to arterial shear function. Future studies will continue to explore these relationships and further define mechanisms of TIC.

## Supporting information

S1 Table Level I activation requirements. (DOCX)

S1 Fig Bleeding times in polytrauma vs. minor trauma patient samples. Microfluidic bleeding times at venous and arterial shear comparing polytrauma (ISS≥16) vs. minor trauma (ISS<16).
(TIF)

S2 Fig Hematology parameters did not distinguish trauma samples that did or did not achieve microfluidic hemostasis. Complete blood counts were not different in samples separated by those that achieved microfluidic hemostasis (BT<1200 s) or not (BT> = 1200 s). Individual data points are shown with bars at mean. ns: not significant * p<0.05, ** p<0.01, *** p<0.001.
(TIF)

S3 Fig Plasma-based assays largely do not correlate with the same signatures as microfluidic parameters. Correlations shown with prothrombin time (PT) and partial thromboplastin time (PTT) with the same parameters identified in the correlation analysis as having significant relationships with microfluidic bleeding times and growth rate.
(TIF)
