# Supplementary material for: Trauma patients have reduced ex vivo flow-dependent platelet hemostatic capacity in a microfluidic model of vessel injury
Source: PLoS One. 2024 Jul 10;19(7):e0304231. doi: 10.1371/journal.pone.0304231 (PMC11236159; doi:10.1371/journal.pone.0304231)
Supplement: S1 Table — (DOCX) [file pone.0304231.s001.docx]

**S1 Table. Level I Activation Requirements**.

| **Barnes Jewish Hospital/Washington University in St. Louis Level I Activation Requirements** |
| --- |
| Glasgow Coma Scale <14 (at time of report) |
| Systolic blood pressure <90 |
| Respiratory rate <10 or >29 |
| *OR:* Airway compromise or obstruction, flail chest, hemo or pneumothorax, patients intubated prehospital. |
| Uncontrolled hemorrhage or receiving blood |
| Penetrating injuries to head, neck, T-shirt or boxer short coverage areas. |
| Extremity trauma proximal to wrist or ankle with loss of distal pulse |
| Amputation/near amputation proximal to wrist or ankle |
| Paralysis or signs of spinal cord or cranial nerve injury (temporal bone fx) |
| Major burns of ≥ 20% BSA |
| Any signs of inhalation injury |
| Electrical injury ≥ 200 volts (does not include Taser injury) |
| Two or more long-bone fractures (humerus/femur) |
| Pelvic fractures (known on arrival) |
| Open or depressed skull fractures (known on arrival) |

Listed are the institution-specific requirements for Level I activation. Patients must meet one requirement to be elevated to Level I.
