# Supplementary figures and images for: Trauma patients have reduced ex vivo flow-dependent platelet hemostatic capacity in a microfluidic model of vessel injury
Source: PLoS One. 2024 Jul 10;19(7):e0304231. doi: 10.1371/journal.pone.0304231 (PMC11236159; doi:10.1371/journal.pone.0304231)

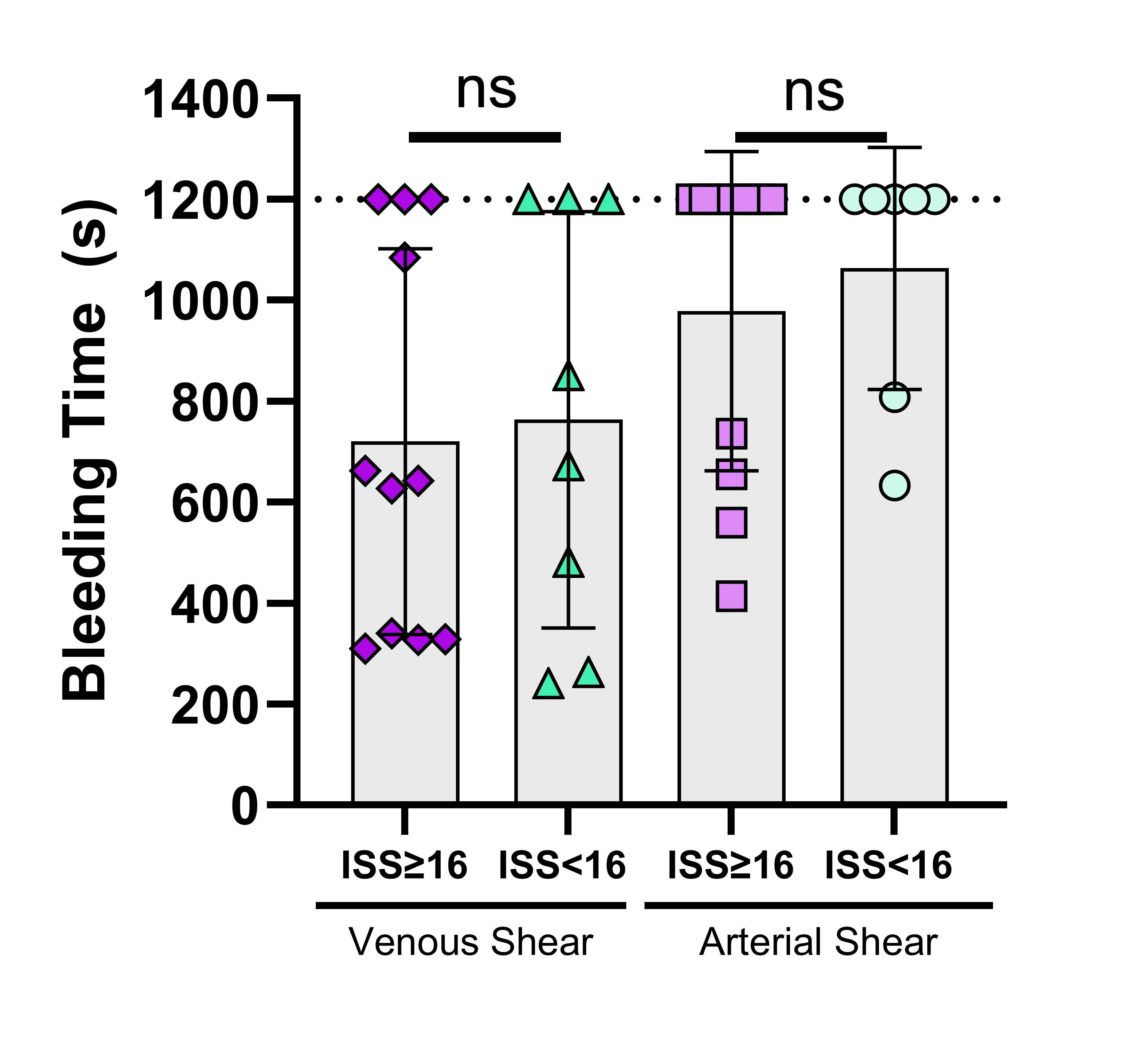

Supplement: S1 Fig — Microfluidic bleeding times at venous and arterial shear comparing polytrauma (ISS≥16) vs. minor trauma (ISS<16). (TIF) [file pone.0304231.s002.tif]
